# NLRP3 inhibition attenuates early brain injury and delayed cerebral vasospasm after subarachnoid hemorrhage

**DOI:** 10.1186/s12974-021-02207-x

**Published:** 2021-07-20

**Authors:** William S. Dodd, Imaray Noda, Melanie Martinez, Koji Hosaka, Brian L. Hoh

**Affiliations:** Department of Neurosurgery, College of Medicine, University of Florida, Gainesville, FL 32610 USA

**Keywords:** Subarachnoid hemorrhage, Microglia, NLRP3 inflammasome, Stroke, Inflammation

## Abstract

**Background:**

The NLRP3 inflammasome is a critical mediator of several vascular diseases through positive regulation of proinflammatory pathways. In this study, we defined the role of NLRP3 in both the acute and delayed phases following subarachnoid hemorrhage (SAH). SAH is associated with devastating early brain injury (EBI) in the acute phase, and those that survive remain at risk for developing delayed cerebral ischemia (DCI) due to cerebral vasospasm. Current therapies are not effective in preventing the morbidity and mortality associated with EBI and DCI. NLRP3 activation is known to drive IL-1β production and stimulate microglia reactivity, both hallmarks of SAH pathology; thus, we hypothesized that inhibition of NLRP3 could alleviate SAH-induced vascular dysfunction and functional deficits.

**Methods:**

We studied NLRP3 in an anterior circulation autologous blood injection model of SAH in mice. Mice were randomized to either sham surgery + vehicle, SAH + vehicle, or SAH + MCC950 (a selective NLRP3 inhibitor). The acute phase was studied at 1 day post-SAH and delayed phase at 5 days post-SAH.

**Results:**

NLRP3 inhibition improved outcomes at both 1 and 5 days post-SAH. In the acute (1 day post-SAH) phase, NLRP3 inhibition attenuated cerebral edema, tight junction disruption, microthrombosis, and microglial reactive morphology shift. Further, we observed a decrease in apoptosis of neurons in mice treated with MCC950. NLRP3 inhibition also prevented middle cerebral artery vasospasm in the delayed (5 days post-SAH) phase and blunted SAH-induced sensorimotor deficits.

**Conclusions:**

We demonstrate a novel association between NLRP3-mediated neuroinflammation and cerebrovascular dysfunction in both the early and delayed phases after SAH. MCC950 and other NLRP3 inhibitors could be promising tools in the development of therapeutics for EBI and DCI.

**Supplementary Information:**

The online version contains supplementary material available at 10.1186/s12974-021-02207-x.

## Introduction

Inflammasomes are innate immune regulatory elements that drive the sterile inflammation characteristic of many disease states. In the presence of damage-associated molecular patterns (DAMPs) and other stimuli, inflammasomes are responsible for the proliferation of the immune response via regulating the post-translational processing of proinflammatory cytokines [1]. This action makes inflammasomes essential mediators of inflammatory diseases as well as ideal drug targets as their inhibition can prevent signal transduction and propagation early in the inflammatory cascade. Activation of the NLRP3, NOD2, and AIM2 inflammasomes, as well as the related innate immune-stimulating cGAS-STING pathway, has all been described in the context of cerebrovascular disease [2–4]. The NLRP3 inflammasome is increasingly recognized as especially important in neurological and cardiovascular disease due to its unique expression profile and activation pattern [5, 6]. NLRP3 is expressed in myeloid cells and complexes with ASC and caspase-1 upon activation to regulate the expression of IL-1β, IL-18, and gasdermin D (GSDMD) [7]. In vitro experiments have isolated NLRP3 activators that are common DAMPs in vascular disease, such as cholesterol crystals, heme, oxidized lipids, ATP, and mtDNA [1]; however, many context- and disease-specific consequences of inflammasome activation remain unexplored despite potentially massive value in the advancement of clinical practice.

Subarachnoid hemorrhage (SAH) is a devastating illness with poor outcomes and disproportionately high mortality [8]. Approximately 35% of deaths from SAH will occur within 48 h of the ictal event [9], and about one-third of survivors will develop long-term neurological and cognitive deficits (referred to as delayed cerebral ischemia) [10]. Pathophysiologically, the poor outcomes are believed to result from a combination of cerebral edema, formation of microthrombi, neuroinflammation, and delayed cerebral vasospasm [11]. Each of these factors has been independently linked to poor outcomes and neuronal cell death following SAH [12–15], but potential therapeutics targeting vasculature directly have had mixed results in clinical trials and can worsen cardiopulmonary status [16]. Innovative therapeutic strategies are urgently needed.

The evidence for neuroinflammation as a key mediator of cerebrovascular dysfunction and poor outcomes after SAH continues to mount [17]. Depletion of microglia or Ly6G/C^+^ cells and genetic deletion of TLR4 or MyD88 have been shown to prevent vasospasm and neuronal apoptosis following SAH [18, 19], supporting the notion that inflammatory cells and pathways have a fundamental role in SAH-related vascular pathology. The NLRP3 inflammasome is expressed in microglia [20] and known to activate in the presence of free heme molecules [1], but the precise relationship between NLRP3 and cerebrovascular dysfunction following SAH remains unclear. A recent report by Hirsch and colleagues demonstrated that caspase-1 activity is elevated in SAH patients and correlates with functional outcomes [21], emphasizing the need for further investigation into the NLRP3 pathway. We hypothesized that NLRP3 inhibition would prevent inflammasome activation, dampen microglial reaction, and ameliorate vascular dysfunction after SAH.

## Materials and methods

### Animals

All animal experiments were performed in accordance with our IACUC (Institutional Animal Care and Use Committee) and ARRIVE (Animal Research: Reporting of In Vivo Experiments) guidelines. All animals were 12–15-week-old female C57BL/6 mice and were housed in pathogen-free housing with ad libitum food and water and 12-h light/dark cycling. All mice were received from Charles River Labs (Wilmington, MA). All animals underwent twice daily monitoring from institutional animal care services staff and/or investigators. We utilized a block randomization scheme wherein animals were randomized by cage number to treatment groups prior to surgery.

### Subarachnoid hemorrhage model

SAH was modeled using a modified anterior circulation single autologous blood injection method [22, 23]. Mice were anesthetized with ketamine (100 mg/kg) and xylazine (10 mg/kg) before removing hair from scalp and cleaning with serial betadine–saline washes. The tail was also prepped from the base extending 1cm distally. Mice were then fixed into a stereotaxic frame and body temperature maintained at 37°C through an adjustable heating pad. The scalp was incised along the sagittal suture from bregma to the nasal bone. The skin was reflected, and a burr hole was drilled 5.0-mm rostral of bregma and 0.5mm right laterally to avoid puncturing the dural venous sinuses. Next, 150μL of arterial blood was drawn from the ventral tail artery onto paraffin wax paper, and hemostasis was achieved with manual pressure using sterile gauze. Fifty microliters of blood was drawn into a Hamilton syringe and passed through the cranial burr hole at an angle of 30° caudally until contact with the skull base (approximately 7.2mm). The syringe was left in place for 30 s before the blood was manually injected at a rate of 10μL/min (Supplemental Fig. 1A). After blood injection was complete, the syringe was left in place for an additional 3 min and then retracted slowly at a rate of 1 mm/min. Sham surgery was the identical procedure and timing including placement of the stereotactic needle but without blood injection (Supplemental Fig. 1B). After the needle and syringe were removed, the burr hole was covered, and skin flap was sutured with 5-0 ethilon suture.

### Pharmaceutics and dosing

MCC950, a selective inhibitor of NLRP3 [24], was obtained from Selleckchem (Houston, TX) and diluted in saline. Saline was used as vehicle control for all experiments. MCC950 (40 mg/kg) or an equivalent volume of vehicle was delivered via intraperitoneal injection 15 min post-SAH. For experiments ending at 24 h post-SAH, an identical dose of MCC950 or vehicle was administered again 12 h later. For experiments ending 5 days post-SAH, MCC950 or vehicle was administered again on days 1 and 3 post-SAH (Supplemental Fig. 1C). Dosage and dosing strategy were selected based on previous studies targeting the vasculature and ischemic stroke [25, 26].

### Immunofluorescence and TUNEL assay

Following perfusion-fixation and overnight post-fixation in 4% PFA, brain samples were embedded in paraffin wax and cut into 7-μm sections with a Leica RM2125 RTS microtome (Leica Microsystems, Buffalo Grove, NY) and adhered to poly-l-lysine-coated glass slides. Serial sections were deparaffinized in mixed xylenes (2 × 3 min, 27 °C) and rehydrated in graded ethanol solutions and deionized water. Antigen retrieval was performed by incubating slides in Dako Target Retrieval Solution, pH 9 (Agilent, Santa Clara, CA) at 95 °C for 20 min. Slides were then washed with Tris-buffered saline with 0.05% Tween-20 (TBST) before being incubated overnight with specified primary antibodies (Table 1) or isotype control. Primary antibodies were detected using donkey anti-rabbit IgG AlexaFluor594 (ThermoFisher, Waltham, MA). FITC-conjugated dUTP-based TUNEL kit was obtained from Abcam (Cambridge, MA) and performed according to manufacturer’s instructions on NeuN-stained sections. Slides were mounted with a DAPI-containing antifade mounting medium (VectaShield, Vector Labs, Burlingame, CA) and imaged using an Olympus IX71 fluorescent microscope (Olympus America, Center Valley, PA). All images were analyzed by two observers blinded to group assignment.

**Table 1:** Antibodies utilized with source, catalog number, and dilution

Antibody	Source (catalog #)	Dilution (rel. to 1mg/mL stock)
Mouse anti-GAPDH	Abcam (ab8245)	1:10,000
Anti-mouse IgG (HRP-conj.)	Abcam (ab205729)	1:25,000
Anti-rabbit IgG (HRP-conj.)	Abcam (ab6721)	1:25,000
Rabbit anti-NLRP3	Bioss (BS-10021R)	1:2000
Rabbit anti-caspase-1 (p10)	Abcam (ab1795015)	1:1000
Mouse anti-capase-1 (p20)	Adipogen (AG-20B-0042-C100)	1:1000
Rabbit anti-IL-1β	Abcam (ab9722)	1:1000
Rabbit anti-ZO-1	ThermoFisher (61-7300)	1:2000
Mouse anti-occludin	ThermoFisher (OC-3F10)	1:1000
Rabbit anti-Iba1	Wako (019-19741)	1:1000
Mouse anti-Iba1	ThermoFisher (MA5-27726)	1:100
Rabbit anti-NeuN	Millipore Sigma (ABN78)	1:500
Rat anti-NIMP-14 (Ly6G/C)	Abcam (ab2557)	1:200
Rabbit anti-fibrinogen β-chain	ThermoFisher (16747-1-AP)	1:100

### Microglia morphology analysis

Five 40× images from Iba1-stained slides of each sample were taken at random points in the ipsilateral cerebral cortex by an observer blinded to treatment. Individual Iba1^+^ cells with identifiable cell bodies were then processed through the ImageJ software according to previously established protocols [27] to generate the number of process endpoints for each cell. The blinded observer also counted the total number of microglia per field for all images to quantify the total microglia burden.

### Brain water content measurements

Brain water content was measured using the wet weight–dry weight method [28]. Twenty-four hours post-SAH, brains were removed from the skull and cut along the midsagittal plane to isolate the ipsilateral (right) hemisphere. The olfactory lobe and hindbrain were removed from the cortex and midbrain. Brains were weighed, placed into a 60°C oven, and allowed to dry for 48 h. Dried brain tissue was then reweighed and used to calculate the percent water content.

### Western blotting

At 24 h or 7 days post-SAH, mice were deeply anesthetized and perfused with 10mL of ice-cold PBS. Brains were harvested from the skull and placed into a cold brain matrix (Harvard Apparatus, Holliston, MA). The olfactory lobe and hindbrain were removed, and brains were cut in half along the mid-sagittal plane. The right cortex was then cut again 3mm caudally from the rostral edge, and the pieces of tissue were snap frozen in liquid N_2_. Once all tissue samples were collected, brains were homogenized with a Dounce homogenizer and incubated in RIPA buffer at 4°C for 90 min with constant agitation. Samples were centrifuged, supernatant collected, and protein quantified via Bradford assay. Samples were then frozen at −20°C or used immediately. Thirty-five micrograms of total protein was separated electrophoretically through a 4–15% tris-glycine gel at 90V for 60 min and transferred onto nitrocellulose or PVDF membranes with 0.45-μm pore size at 90V for 30 min. Membranes were blocked with 5% skim milk in TBST for 1 h at room temperature and then probed with specified primary antibodies (Table 1) diluted in 5% skim milk overnight at 4°C with constant agitation. Membranes were then washed three times with TBST and then incubated with HRP-conjugated secondary antibody diluted in 5% skim milk for 1 h at room temperature with constant agitation. Finally, membranes were washed five times with TBST and incubated with luminol-peroxide solution for 45 s before being exposed to film. Films were developed, and densitometry performed using the ImageJ software. Band intensities were normalized to loading control (GAPDH) to generate a target/GAPDH ratio and normalized to sham + vehicle group.

### Cerebral vasospasm measurements

Middle cerebral artery diameter was measured as previously reported [29, 30]. Five or seven days post-SAH, mice were deeply anesthetized with ketamine and xylazine before being serially perfused through the left ventricle with PBS (5mL), 4% paraformaldehyde (15mL), and 20% Indian ink dissolved in 5% gelatin. All solutions were administered at a constant rate of 15 mL/min to mimic cardiac output [31]. Mouse carcasses were then stored at 4 °C for 8 h to allow for gelatin hardening. Brains were imaged and analyzed by an observer blinded to group assignment. The narrowest diameter within the M1 segment of the middle cerebral artery (MCA) was measured to assess vasospasm. Mice that were anatomic variants of the standard circle of Willis (e.g., bifurcated/duplicated M1s, aplastic P1s, bilateral hypoplastic MCAs) were excluded from analysis.

### Neurological evaluation

Mice were observed and score for gross neurological deficits using a composite grading system developed for murine models of subarachnoid hemorrhage by Sugawara et al. [32]. The scoring components and descriptions are outlined in Table 2. Additionally, we used the corner test [33, 34] to detect sensorimotor deficits specific to injury in the areas affected by our model (M1 vascular distribution). Two 20-cm long boards were adjoined at a 30° angle and placed in a clean animal cage. One to three days prior to the SAH procedure, the mice were placed in the behavioral testing environment for 10 min and allowed to acclimate. At the end of the 10-min acclimatization period (pre-SAH) and then again on day 2, day 3, and day 5 post-SAH, each mouse was placed between the two boards, half way to the corner. The turning direction after the mouse entered deep into the corner was recorded for two trials of ten turns each. Only turns that were part of a rearing movement (i.e., the forepaws lift and touch the sidewalls during the turn) were counted.

**Table 2:** Composite neurological score system

Test	Score			
	0	1	2	3
**Spontaneous activity (in cage for 5 min)**	**No movement**	**Barely moves position**	**Moves but does not approach at least three sides of cage**	**Moves and approaches at least three sides of cage**
**Spontaneous movements of all limbs**	**No movement**	**Slight movement of limbs**	**Moves all limbs but slowly**	**Move all limbs same as pre-SAH**
**Movements of forelimbs (outstretching while held by tail)**	**No outreaching**	**Slight outreaching**	**Outreach is limited and less than pre-SAH**	**Outreach same as pre-SAH**
**Climbing wall of wire cage**		**Fails to climb**	**Climbs weakly**	**Normal climbing**
**Reaction to touch on both side of trunk**		**No response**	**Weak response**	**Normal response**
**Response to vibrissae touch**		**No response**	**Weak response**	**Normal response**

### Data reporting and statistical methods

All data are presented as mean ± standard error of the mean (SEM) unless otherwise noted. Non-parametric Kruskal-Wallis test with Dunn’s multiple comparisons test was used to compare the three groups in each experiment. Two-way ANOVA with Tukey’s multiple comparisons test was used to compare composite neurological scores and corner test data across time and condition. Power calculations were completed for the delayed vasospasm experiment with an α of 0.05, β of 0.2 to detect a minimum biological difference of 20% between groups, and predicted standard deviation of 15% MCA diameter. All data analysis was performed using the Prism data analysis software (GraphPad Software, San Diego, CA).

## Results

### MCC950 inhibits NLRP3 inflammasome activation 24 h post-SAH

**Fig. 1:**
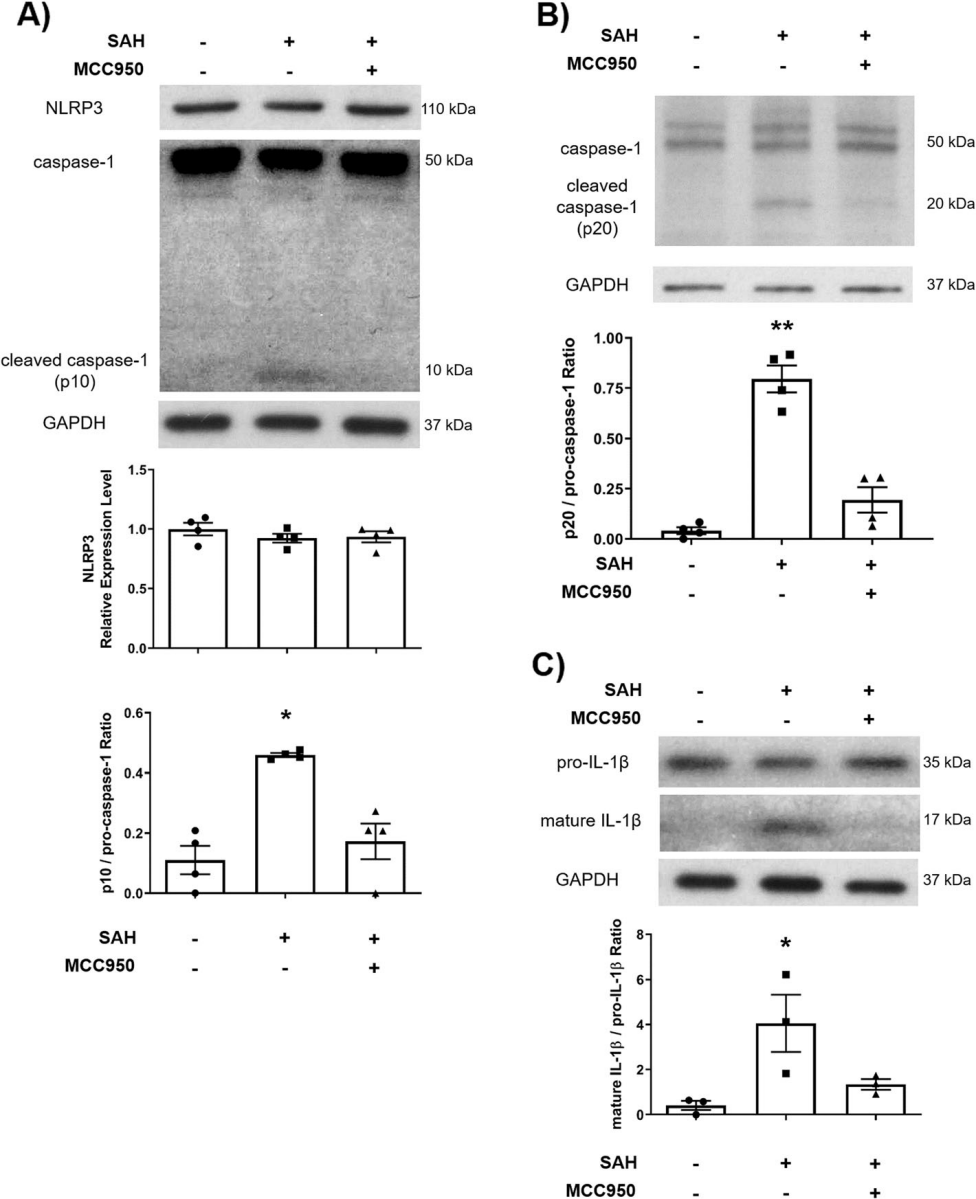
MCC950 treatment inhibits NLRP3 inflammasome activation 24 h post-SAH. **A** Representative western blot and densitometric analysis for NLRP3 and caspase-1 (p10 subunit). **B** Representative western blot and densitometric analysis of caspase-1 (p20 subunit). **C** Representative western blot and densitometric analysis for IL-1β. Data presented as mean ± SEM, * *p* < 0.05 compared to sham + vehicle group, ** *p* < 0.01 compared to sham + vehicle group by Kruskal-Wallis test with Dunn’s multiple comparisons test

We first sought to confirm the efficacy of MCC950-mediated NLRP3 inhibition in our SAH model. We hypothesized that NLRP3 inhibition after SAH would decrease the relative abundance of cleaved caspase-1 as well as reduce mature IL-1β production. We measured NLRP3, caspase-1, and IL-1β protein levels via immunoblotting of ipsilateral cortical brain homogenate at 1-day post-SAH. NLRP3 was unchanged in all groups (sham + vehicle 1.00 ± 0.05, SAH + vehicle 0.92 ± 0.04, SAH + MCC950 0.94 ± 0.05 relative expression units); however, the cleaved caspase-1 p10 and p20 subunits were both increased in the SAH + vehicle group (p10: sham + vehicle 0.11 ± 0.05, SAH + vehicle 0.46 ± 0.01, SAH + MCC950 0.17 ± 0.06 p10/pro-caspase-1 ratio; p20: sham + vehicle 0.04 ± 0.02, SAH + vehicle 0.80 ± 0.07, SAH + MC950 0.19 ± 0.06 p20/pro-caspase-1 ratio) (Fig. 1 A and B). Further, IL-1β, which is cleaved to active form by activated caspase-1, was also increased after SAH but limited by MCC950 treatment (mature IL-1β/GAPDH ratio 0.02 ± 0.01 (sham) vs 0.16 ± 0.04 (SAH + vehicle) vs 0.07 ± 0.02 (SAH + MCC950)) (Fig. 1 C). These findings confirm MCC950 treatment is capable of effectively inhibiting NLRP3 inflammasome activation after subarachnoid hemorrhage.

### NLRP3 inhibition prevents microglia morphology shift after SAH

**Fig. 2:**
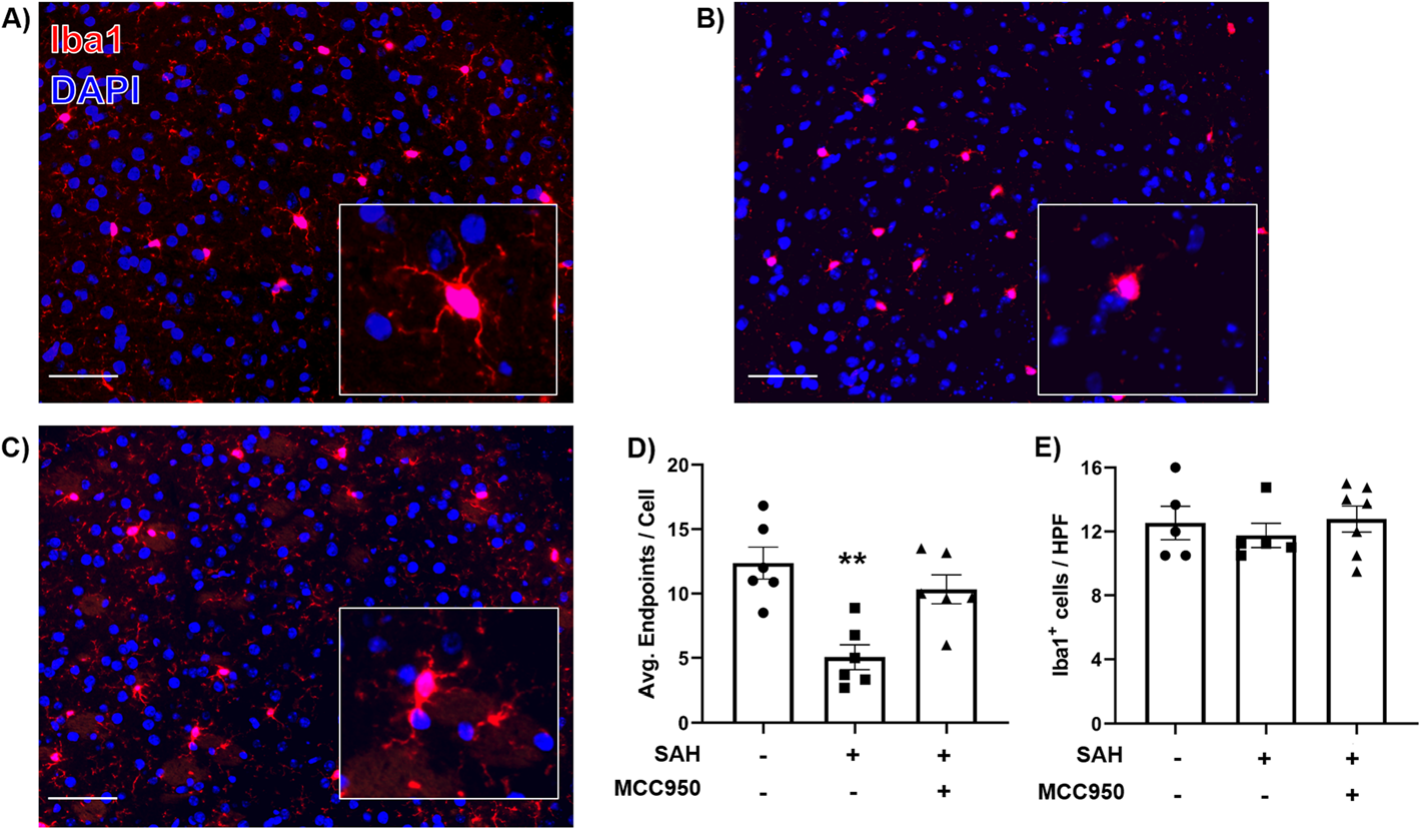
NLRP3 inhibition with MCC950 prevents microglia morphology shift after SAH. **A**–**C** Representative images of Iba1-stained (red) cerebral cortex in **A** sham, **B** SAH + vehicle, and **C** SAH + MCC950 groups with DAPI nuclear counterstain (blue). Scale bars = 50μm, all images captured with 40× objective. Inset: Enlarged images of individual cell bodies. **D** Microglia morphology analysis via quantification of ramification endpoints per cell. **E** Total number of Iba1+ cells per high-powered field as a measurement of microglial burden. Data presented as mean ± SEM, *n* = 5–6 per group for all data, ** *p* < 0.01 compared to sham surgery group by Kruskal-Wallis test with Dunn’s multiple comparisons test

Microglia are well-known to adopt a morphologic shift from ramified to amoeboid upon reacting to stroke injury [35]. We assessed the effect of NLRP3 inhibition on microglia morphology by automated counting of the number of endpoints of Iba1^+^ cell bodies in the cerebral cortex 24 h post-SAH. SAH surgery caused a significant decrease in endpoints (sham 12.37 ± 1.24 vs SAH + vehicle 5.05 ± 0.97 endpoints/cell) (Fig. 2 A and B). MCC950 treatment blunted this response (10.33 ± 1.12 endpoints/cell) (Fig. 2 C). Total microglial burden in the ipsilateral cerebral cortex was unchanged in all groups (sham + vehicle 12.53 ± 1.05, SAH + vehicle 11.75 ± 0.76, SAH + MCC950 12.79 ± 0.81 Iba1^+^ cells/HPF) (Fig. 2 E). These results indicate NLRP3 inhibition prevents microglial morphology shift without affecting the number of microglia present.

### NLRP3 inhibition reduces early brain injury after SAH

**Fig. 3:**
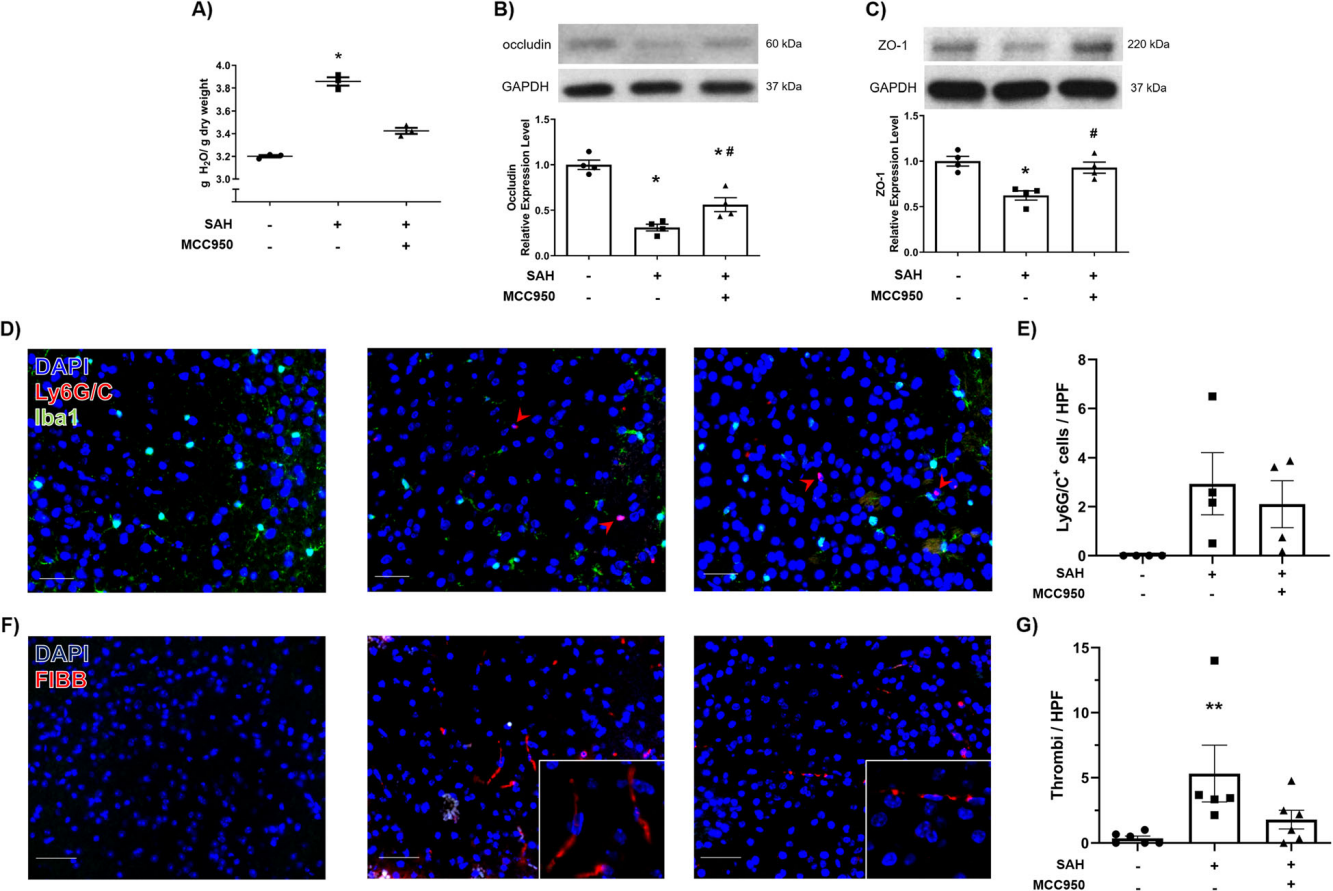
NLRP3 inhibition reduces early brain injury after SAH. **A** Brain water content measurements of the cerebral cortex 24 h post-SAH, *n* = 3 per group. **B**, **C** Western blot for transmembrane tight junction protein occluding (**B**) and ZO-1 (**C**) 24 h post-SAH, *n* = 4 per group. **D** Immunofluorescence staining for infiltrating neutrophils (red) 24 h post-SAH with Iba1 co-stain (green) to distinguish microglia in sham (left), SAH + vehicle (middle), and SAH + MCC950 (right) groups with DAPI nuclear counterstain (blue), scale bars = 50μm, red arrowheads indicate individual neutrophils. **E** Quantification of neutrophils (Ly6G/C^+^ cells) per HPF, *n* = 4 per group. **F** Representative images of fibrinogen-stained (red) cerebral cortex images captured with 40× or 64× (inset) objective lens. **G** Quantification of microthrombi per high-powered field (40×), *n* = 5–6 per group. All data presented as mean ± SEM, * *p* < 0.05, ** *p* < 0.01, compared to sham surgery group, # *p* < 0.05 compared to SAH + vehicle group by Kruskal-Wallis test with Dunn’s multiple comparisons test

Cerebral edema, tight junction disruption, and peripheral immune cell infiltration are characteristic components of early brain injury. We assayed these parameters to evaluate the role of NLRP3 inflammasome in the early phase of SAH pathology. MCC950 partially reduced the development of cerebral edema 24 h post-SAH (sham + vehicle 3.20 ± 0.01, SAH + vehicle 3.86 ± 0.04, SAH + MCC950 3.43 ± 0.03 g H_2_O/g dry weight) (Fig. 3 A). Further, MCC950 preserved the expression of the transmembrane tight junction protein occludin (sham + vehicle 1.00 ± 0.05, SAH + vehicle 0.31 ± 0.04, SAH + MCC950 0.56 ± 0.08 relative expression units) and tight junction-associated protein ZO-1 (sham + vehicle 1.00 ± 0.05, SAH + vehicle 0.62 ± 0.05, SAH + MCC950 0.93 ± 0.06 relative expression units) at the same time point (Fig. 3 B and C). We also found that there is an increase in neutrophil (Ly6G/C^+^ cells) infiltration after SAH (sham + vehicle 0.00 ± 0.00 vs. SAH + vehicle 2.94 ± 1.27 Ly6G/C^+^ cells/HPF); however, NLRP3 did not have a strong effect on this phenomenon (2.10 ± 1.92 Ly6G/C^+^ cells/HPF, *p* = 0.06 vs sham + vehicle) (Fig. 3 D). These data support the hypothesis that NLRP3 contributes to development of cerebral edema and blood-brain barrier disruption in the early phase after SAH.

### NLRP3 inhibition prevents microthrombosis after subarachnoid hemorrhage

Microthrombosis is becoming an increasingly appreciated factor in EBI and DCI. Thrombosis in small arteries and arterioles can act in concert with cerebral vasospasm to decrease overall cerebral blood flow [13, 14]. We investigated the effect of NLRP3 inhibition on microthrombosis by immunofluorescent staining of fibrinogen^+^ thrombi in the cerebral cortex 24 h post-SAH. Subarachnoid hemorrhage caused an increase in the number of microthrombi (sham 0.36 ± 0.17 vs SAH + vehicle 5.22 ± 2.2 thrombi/HPF), and MCC950 significantly reduced microthrombi (1.76 ± 0.71 thrombi/HPF) (Fig. 3 E). These findings suggest NLRP3 inhibition is effective at reducing microthrombosis after SAH.

### NLRP3 inhibition reduces neuronal apoptosis 24 h after SAH

**Fig. 4:**
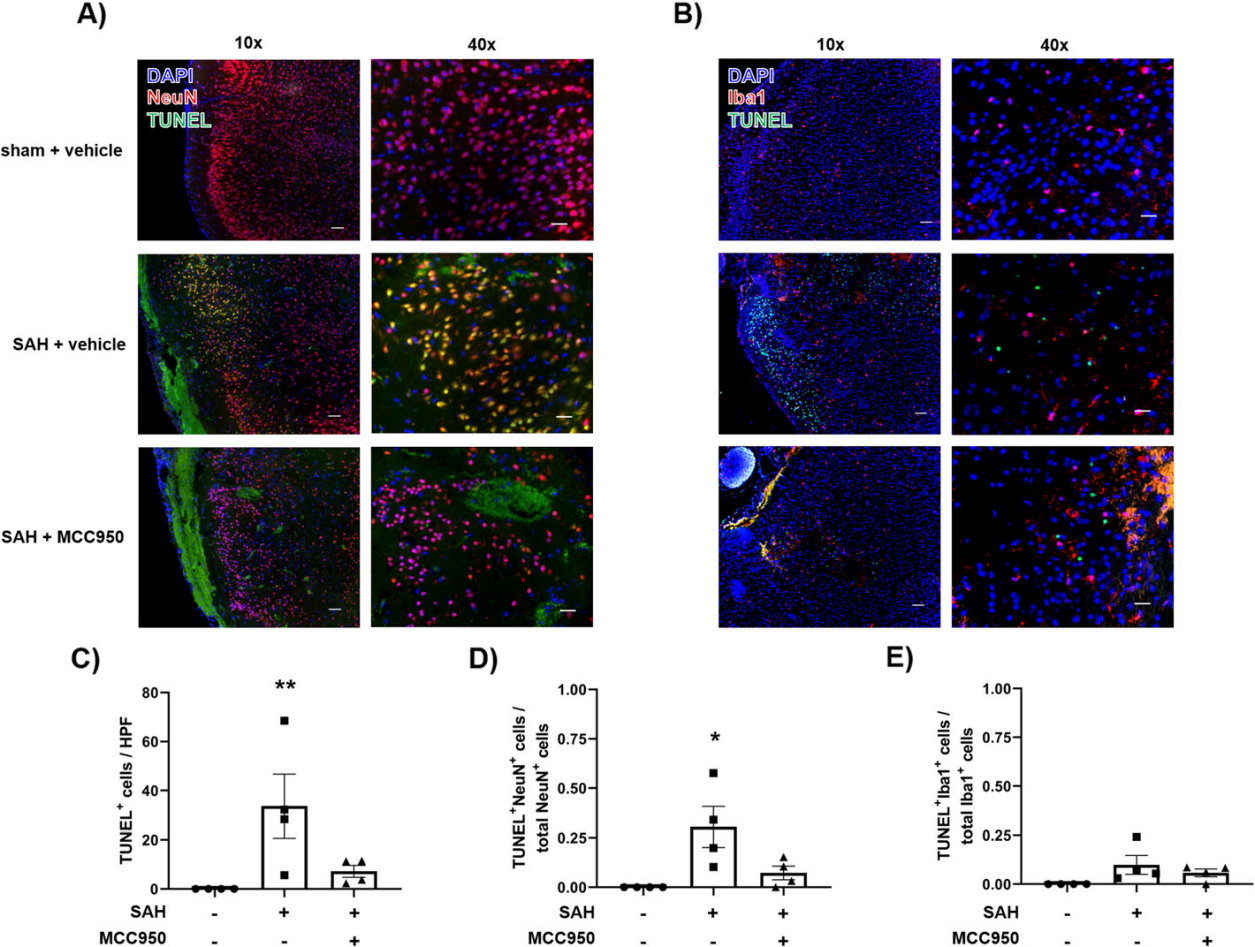
NLRP3 inhibition reduces neuronal apoptosis 24 h after SAH. **A** Representative images of TUNEL assay with NeuN co-stain in sham + vehicle (top), SAH + vehicle (middle), and SAH + MCC950 (bottom). **B** Representative images of TUNEL assay with Iba1 co-stain. **C** Quantification of total TUNEL-positive cells per high-powered field (HPF). **D** Quantification of TUNEL-positive neurons as a proportion of total NeuN-positive cells per HPF. **E** Quantification of TUNEL-positive microglia as a proportion of total Iba1-positive cells per HPF. Images were captured with 10× or 40× objective lens, scale bars = 200μm for 10× images and 50μm for 40× images, *n*= 4 in each group, * *p* < 0.05 compared to sham+ vehicle group, ** *p* < 0.01 compared to sham + vehicle group by Kruskal-Wallis test with Dunn’s multiple comparisons test

Neuronal cell death in the early and delayed phases after SAH likely results in functional neurological deficits in SAH patients. We investigated this aspect of SAH pathology by performing a TUNEL assay with NeuN co-staining. NLRP3 inhibition reduced the total number of TUNEL^+^ cells in the cerebral cortex (SAH + vehicle 24.16 ± 5.48 vs. SAH + MCC950 5.99 ± 3.10 TUNEL^+^ cells/HPF) and reduced the proportion of neurons showing TUNEL positivity (SAH + vehicle 30.45 ± 10.30% vs. SAH + MCC950 7.51 ± 3.30% TUNEL^+^ neurons/HPF) (Fig. 4). We also investigated programmed cell death in microglia by performing the TUNEL assay with Iba1 co-stain. Compared to neurons, relatively few microglia were TUNEL^+^ after SAH, and inhibition of the NLRP3 pathway had no effect (sham + vehicle 0.00 ± 0.00%, SAH + vehicle 8.93 ± 4.93%, SAH + MCC950 6.94 ± 3.54% TUNEL^+^ microglia/HPF) (Fig. 4).

### NLRP3 inhibition decreases delayed cerebral vasospasm and sensorimotor deficits after SAH

**Fig. 5:**
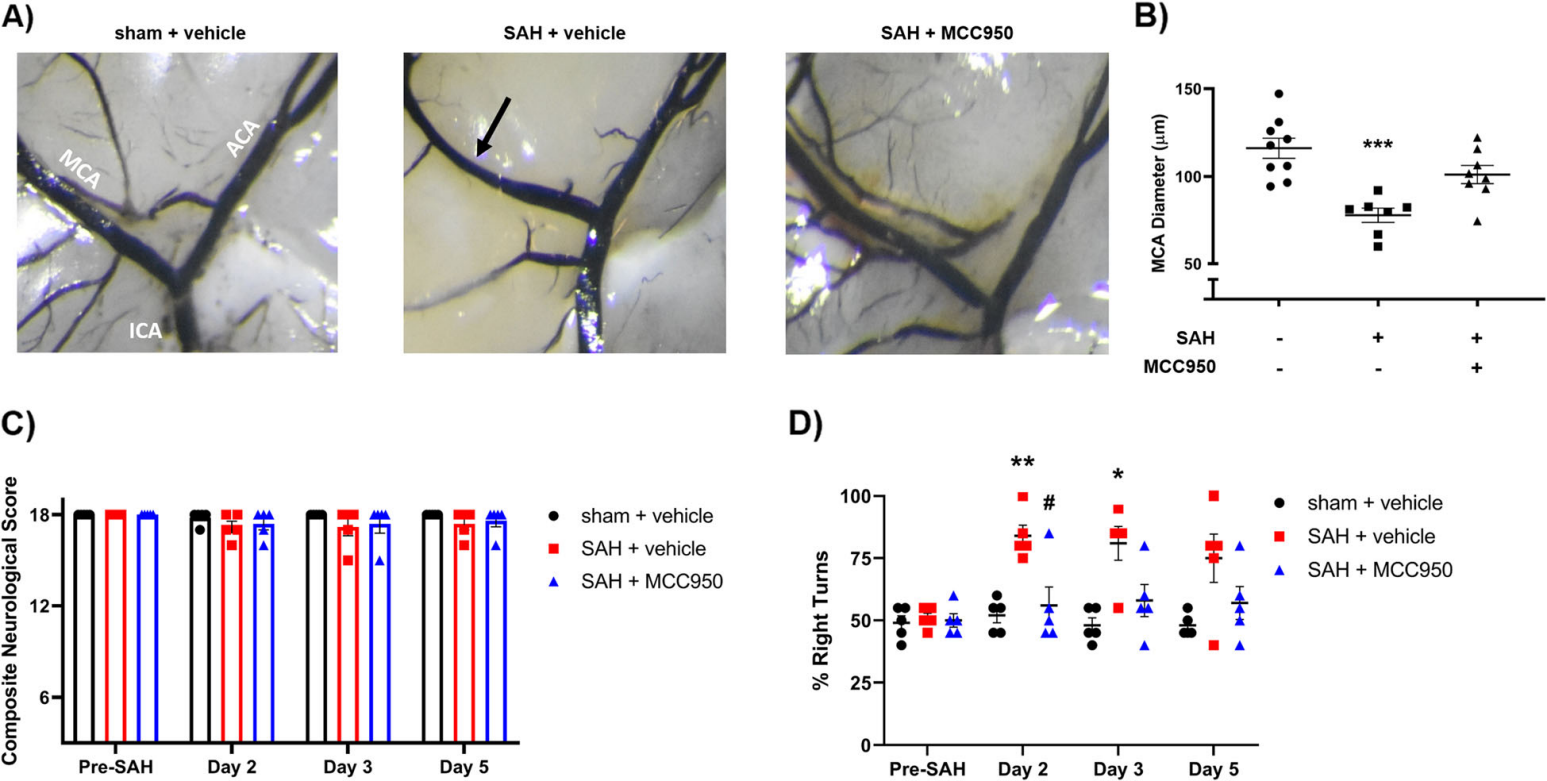
NLRP3 inhibition decreases delayed cerebral vasospasm after SAH. **A** Representative images of vessel-casted brains of sham + vehicle (left), SAH + vehicle (middle), and SAH + MCC950 (right) groups 5 days after SAH. The internal carotid artery (ICA), middle cerebral artery (MCA), and anterior cerebral artery (ACA) are identified in sham image. Arrows indicate areas of significant vasospasm, scale bars = 100μm. **B** Middle cerebral artery measurements normalized to sham group. Data presented as mean ± SEM, *n* = 7–9 per group, *** *p* < 0.001 compared to sham surgery group by Kruskal-Wallis test with Dunn’s multiple comparison test. **C** Composite neurological evaluation score, *p* > 0.05 at all timepoints and across all groups by two-way ANOVA. **D** Prevalence of right turns in the corner test, * *p* < 0.05, ** *p* < 0.01 vs sham + vehicle group, # *p* < 0.05 vs SAH + vehicle group by two-way ANOVA with Tukey’s multiple comparison test

The development of DCI is a leading contributor to unfavorable outcomes after subarachnoid hemorrhage. Delayed cerebral vasospasm is a significant contributor to DCI by further restricting cerebral blood flow [15]. We hypothesized that NLRP3 inhibition via MCC950 would prevent cerebral vasospasm. We measured ipsilateral MCA diameter 5 days post-SAH in mice treated with either vehicle or MCC950 and found that MCC950 significantly reduced the severity of vasospasm (sham + vehicle 116.2 ± 5.7 μm, SAH + vehicle 77.8 ± 4.1 μm, SAH + MCC950 101.1 ± 5.2 μm) (Fig. 5 A and B). A separate cohort of mice was sacrificed at 7 days post-SAH to both investigate the durability of vasospasm and observe a timepoint closer to that at which humans begin to develop cerebral vasospasm. NLRP3 inhibition with MCC950 remained effective at preventing vasospasm at this timepoint as well (Supplemental Fig. 2).

We also evaluated the functional outcomes with a composite neurological exam score (Table 2) and the corner test. We found no change in composite neurological score at any timepoint (Fig. 5 C); however, mice in the SAH + vehicle group were significantly more likely to make ipsilateral turns in the corner test. NLRP3 inhibition with MCC950 prevented this phenomenon and relieved the functional deficit (Fig. 5 D).

## Discussion

Brain injury after SAH occurs via a biphasic time-course characterized by two phases of distinctive pathologic mechanisms. The first early phase, EBI, is defined by transient global ischemia during the ictal event, cerebral edema, blood-brain barrier breakdown, and microthrombosis. The delayed phase is characterized by vasospasm of the cerebral arteries and apoptosis of neurons, leading to the development of DCI. Current therapies have only limited efficacy to prevent DCI [16]. In this study, we investigated the role of NLRP3 inflammasome in SAH-induced neuroinflammation [17] and microglial reactivity [19, 36] as a contributor to vascular dysfunction after SAH. We found NLRP3 inhibition prevented both the increased IL-1β expression and the microglia morphology shift typical of hemorrhagic stroke. Further, we demonstrated NLRP3 inhibition successfully attenuated the characteristic cerebrovascular dysfunction following SAH: cerebral edema, tight junction disruption, microthrombosis, neuronal apoptosis, and delayed cerebral vasospasm. Finally, we showed these results are functionally significant through preventing the development of sensorimotor deficits in the corner test. These findings both elucidate the interaction between neuroinflammation and cerebrovascular dysfunction and form the framework in which MCC950 and other NLRP3 inhibitors can be further studied as a candidate therapy for SAH patients.

Although all cell types in the CNS contribute to inflammatory responses [37], microglia are generally understood to be one of the primary sources of proinflammatory cytokines in most cases [38]. Microglia, but not astrocytes, express all the components of the NLRP3 inflammasome and produce IL-1β [20]. Our findings that NLRP3 inhibition suppresses IL-1β production and microglia morphology shift (Fig. 1 and 2) lead us to hypothesize that microglia are the primary source of NLRP3-caspase-1 pathway activation after SAH and MCC950 can suppress microglial-mediated neuroinflammation in this context. Importantly, MCC950 did not change the total number of microglia present in the cerebral cortex (Fig. 2 E), and few microglia underwent programmed cell death after SAH (Fig. 4). The role of microglia after CNS injury is multifaceted, and microglia are necessary to phagocytize cellular debris and regulate the reestablishment of neuronal connections [38, 39]. NLRP3 inhibition and IL-1β reduction without reducing microglia availability may represent an optimal outcome following SAH and contribute to the preservation of cerebrovascular function.

In this study, we measured a number of cerebrovascular outcomes after SAH. This is particularly important as DCI and the associated poor outcomes in SAH patients are due to a confluence of vascular disturbances rather than any single variable alone [13, 40]. By studying NLRP3’s effect on cerebral edema, tight junctions, microthrombosis, and delayed cerebral vasospasm (Figs. 3 and 5), we can more confidently characterize the NLRP3 pathway as an important avenue of future research. Multiple experiments by different investigators have demonstrated that depletion of microglia reduces vasospasm [19, 36]. Our findings are consistent with the hypothesis that suppression of microglial reactivity is the mechanism by which MCC950 prevents vasospasm after SAH; however, more investigation with cell type-specific manipulations of NLRP3 expression are needed to provide definitive answers. Our data cannot rule out a microglia-independent effect of MCC950 treatment. One such possibility is modulation of neutrophil infiltration, as depletion of Ly6G/C cells has been shown to reduce vasospasm in a murine model. Consistent with those previous data, we found an increase in Ly6G/C cell infiltration after SAH (Fig. 3); however, NLRP3 inhibition seems to affect Ly6G/C cell trafficking only minimally as there remained a trend for increased infiltration after MCC950 administration (Fig. 3).

Our model of subarachnoid hemorrhage does not produce gross neurological deficits and only small changes to composite neurological scores (Fig. 5 E); however, when using a focused functional assessment such as the corner test, we revealed a propensity for ipsilateral turns after subarachnoid hemorrhage (Fig. 5 F). The difference was largest at day 2 post-SAH and gradually returned towards baseline. NLRP3 inhibition via MCC950 treatment prevented an increase in right turn likelihood at all timepoints (Fig. 5 F).

There are some important limitations to consider when drawing conclusions from this study. We used only female mice due to the higher incidence of subarachnoid hemorrhage in women [41]; however, the effect of MCC950 after SAH in males remains to be investigated. Mechanistically, we have associated the effect of NLRP3 inhibition on microglial reactivity with its beneficial effects on the cerebrovasculature but have not shown microglial regulation to be the definitive mechanism by which NLRP3 inhibition ameliorates cerebrovascular dysfunction. Microglia are often cited as the primary source of NLRP3-mediated neuroinflammation [42]; however, because NLRP3 is also expressed by endothelial cells [43, 44] and possibly neurons [45, 46], we cannot rule out the possibility that MCC950 is acting through a different cell type as well. Recent reports on other inflammasome subtypes suggest that brain injury activates AIM2 in neurons [3] and other NLRs in endothelial cells [2]. Further investigation is needed to elucidate the exact mechanism of MCC950’s protective effects, including delineation of the precise role of microglia.

## Conclusions

We have provided a critical link between NLRP3 and cerebrovascular outcomes following SAH. Our study demonstrates that MCC950 has powerful anti-inflammatory effects after SAH that are associated with reduced edema, preserved tight junctions, and amelioration of microthrombosis, delayed cerebral vasospasm, and functional deficits. We propose that MCC950 and the NLRP3 pathway be further studied in the development of treatments for SAH patients.

## Supplementary Information

**Additional file 1: Supplemental Figure 1.** A) Image of sham-operated brain immediately after surgery. B) Image of SAH-operated brain immediately after surgery. C) Experimental timeline.

**Additional file 2: Supplemental Figure 2.** Effect of MCC950 on cerebral vasospasm persists to seven days post-SAH. A-C) Representative images of vessel-casted brains of sham, SAH + vehicle, and SAH + MCC950 groups seven days after SAH. Internal carotid, middle cerebral, and anterior cerebral arteries are identified in sham image. Arrows indicate areas of significant vasospasm, scale bars = 100μm. D) Middle cerebral artery measurements normalized to sham group. Data presented as mean ± SEM, n = 8-9 per group, *** p < 0.001 compared to sham surgery group by Kruskal-Wallis test with Dunn’s multiple comparison.

**Additional file 3.** Raw blot files.

## Data Availability

The datasets used and/or analyzed during the current study are available from the corresponding author on reasonable request.

## Abbreviations

SAH: Subarachnoid hemorrhage
EBI: Early brain injury
DCI: Delayed cerebral ischemia
NLRP3: NOD-, LRR-, and pyrin domain-containing Protein 3
ASC: Apoptosis-associated speck-like protein containing a CARD
BBB: Blood-brain barrier
MCA: Middle cerebral artery

## References

[1] Swanson KV, Deng M, Ting JPY (2019). The NLRP3 inflammasome: molecular activation and regulation to therapeutics. Nat Rev Immunol.

[2] Ge X, Li W, Huang S, Yin Z, Xu X, Chen F, Kong X, Wang H, Zhang J, Lei P (2018). The pathological role of NLRs and AIM2 inflammasome-mediated pyroptosis in damaged blood-brain barrier after traumatic brain injury. Brain Res.

[3] Yuan B, Zhou XM, You ZQ, Xu WD, Fan JM, Chen SJ, Han YL, Wu Q, Zhang X (2020). Inhibition of AIM2 inflammasome activation alleviates GSDMD-induced pyroptosis in early brain injury after subarachnoid haemorrhage. Cell Death Dis.

[4] Li Q, Cao Y, Dang C, Han B, Han R, Ma H, Hao J, Wang L (2020). Inhibition of double-strand DNA-sensing cGAS ameliorates brain injury after ischemic stroke. EMBO Mol Med.

[5] Yang SJ, Shao GF, Chen JL, Gong J (2018). The NLRP3 inflammasome: an important driver of neuroinflammation in hemorrhagic stroke. Cell Mol Neurobiol.

[6] Zhou W (2018). NLRP3: a novel mediator in cardiovascular disease. J Immunol Res.

[7] Rathinam VAK, Fitzgerald KA (2016). Inflammasome complexes: emerging mechanisms and effector functions. Cell.

[8] Wardlaw JM, White PM (2000). The detection and management of unruptured intracranial aneurysms. Brain.

[9] Stienen MN, Germans M, Burkhardt JK, Neidert MC, Fung C, Bervini D, Zumofen D, Roethlisberger M, Marbacher S, Maduri R, Robert T, Seule MA, Bijlenga P, Schaller K, Fandino J, Smoll NR, Maldaner N, Finkenstädt S, Esposito G, Schatlo B, Keller E, Bozinov O, Regli L, Serra C, Krayenbühl N, Schöni D, Raabe A, Beck J, Goldberg J, Mariani L, Guzman R, D’Alonzo D, Coluccia D, Daniel RT, Starnoni D, Messerer M, Levivier M, Valsecchi D, Arrighi M, Venier A, Reinert M, Kuhlen DE, Ferrari A, Weyerbrock A, Hildebrandt G, Hlavica M, Fournier JY, Corniola M, on behalf of the Swiss SOS Study Group* (2018). Predictors of in-hospital death after aneurysmal subarachnoid hemorrhage: analysis of a nationwide database (Swiss SOS [Swiss Study on Aneurysmal Subarachnoid Hemorrhage]). Stroke.

[10] Foreman B (2016). The pathophysiology of delayed cerebral ischemia. J Clin Neurophysiol.

[11] Geraghty JR, Testai FD (2017). Delayed cerebral ischemia after subarachnoid hemorrhage: beyond vasospasm and towards a multifactorial pathophysiology. Curr Atheroscler Rep.

[12] Lublinsky S, Major S, Kola V, Horst V, Santos E, Platz J, Sakowitz O, Scheel M, Dohmen C, Graf R, Vatter H, Wolf S, Vajkoczy P, Shelef I, Woitzik J, Martus P, Dreier JP, Friedman A (2019). Early blood-brain barrier dysfunction predicts neurological outcome following aneurysmal subarachnoid hemorrhage. EBioMedicine.

[13] Terpolilli NA, Brem C, Bühler D, Plesnila N (2015). Are we barking up the wrong vessels?. Stroke.

[14] McBride DW, Blackburn SL, Peeyush KT, Matsumura K, Zhang JH (2017). The role of thromboinflammation in delayed cerebral ischemia after subarachnoid hemorrhage. Front Neurol.

[15] Kassell NF, Sasaki T, Colohan A, Nazar G (1985). Cerebral vasospasm following aneurysmal subarachnoid hemorrhage. Stroke.

[16] Velat GJ, Kimball MM, Mocco JD, Hoh BL (2011). Vasospasm after aneurysmal subarachnoid hemorrhage: review of randomized controlled trials and meta-analyses in the literature. World Neurosurg.

[17] de Oliveira Manoel AL, Loch Macdonald R. Neuroinflammation as a target for intervention in subarachnoid hemorrhage. Front. Neurol. 2018;9:292. 10.3389/fneur.2018.00292. 10.3389/fneur.2018.00292 PMC5941982 29770118

[18] Provencio JJ, Altay T, Smithason S, Moore SK, Ransohoff RM (2011). Depletion of Ly6G/C+ cells ameliorates delayed cerebral vasospasm in subarachnoid hemorrhage. J Neuroimmunol.

[19] Hanafy KA (2013). The role of microglia and the TLR4 pathway in neuronal apoptosis and vasospasm after subarachnoid hemorrhage. J Neuroinflammation.

[20] Gustin A, Kirchmeyer M, Koncina E, Felten P, Losciuto S, Heurtaux T, Tardivel A, Heuschling P, Dostert C (2015). NLRP3 inflammasome is expressed and functional in mouse brain microglia but not in astrocytes. PLoS One.

[21] Hirsch Y, Geraghty JR, Katz EA, Testai FD. Inflammasome caspase-1 activity is elevated in cerebrospinal fluid after aneurysmal subarachnoid hemorrhage and predicts functional outcome. Neurocrit Care. 2020;34(3):889–98. 10.1007/s12028-020-01113-z. 10.1007/s12028-020-01113-z PMC8007683 32996055

[22] Sabri M, Jeon H, Ai J, Tariq A, Shang X, Chen G, Macdonald RL (2009). Anterior circulation mouse model of subarachnoid hemorrhage. Brain Res.

[23] Sabri M, Ai J, Knight B, Tariq A, Jeon H, Shang X, Marsden PA, Macdonald RL (2011). Uncoupling of endothelial nitric oxide synthase after experimental subarachnoid hemorrhage. J Cereb Blood Flow Metab.

[24] Coll RC, Robertson AAB, Chae JJ, Higgins SC, Muñoz-Planillo R, Inserra MC, Vetter I, Dungan LS, Monks BG, Stutz A, Croker DE, Butler MS, Haneklaus M, Sutton CE, Núñez G, Latz E, Kastner DL, Mills KHG, Masters SL, Schroder K, Cooper MA, O'Neill LAJ (2015). A small-molecule inhibitor of the NLRP3 inflammasome for the treatment of inflammatory diseases. Nat Med.

[25] Hong P, Li FX, Gu RN, Fang YY, Lai LY, Wang YW, Tao T, Xu SY, You ZJ, Zhang HF (2018). Inhibition of NLRP3 inflammasome ameliorates cerebral ischemia-reperfusion injury in diabetic mice. Neural Plast.

[26] Ren P (2020). Targeting the NLRP3 inflammasome with inhibitor MCC950 prevents aortic aneurysms and dissections in mice. J Am Heart Assoc.

[27] Young K, Morrison H. Quantifying microglia morphology from photomicrographs of immunohistochemistry prepared tissue using ImageJ. J Vis Exp. 2018;136:e57648. 10.3791/57648. 10.3791/57648 PMC6103256 29939190

[28] Keep RF, Hua Y, Xi G (2012). Brain water content. A misunderstood measurement?. Transl Stroke Res.

[29] Vellimana AK, Zhou ML, Singh I, Aum DJ, Nelson JW, Harris GR, Athiraman U, Han BH, Zipfel GJ (2017). Minocycline protects against delayed cerebral ischemia after subarachnoid hemorrhage via matrix metalloproteinase-9 inhibition. Ann Clin Transl Neurol.

[30] Han BH, Vellimana AK, Zhou M-L, Milner E, Zipfel GJ (2012). Phosphodiesterase 5 inhibition attenuates cerebral vasospasm and improves functional recovery after experimental subarachnoid hemorrhage. Neurosurgery.

[31] Janssen B, Debets J, Leenders P, Smits J (2002). Chronic measurement of cardiac output in conscious mice. Am J Phys Regul Integr Comp Phys.

[32] Sugawara T, Ayer R, Jadhav V, Zhang JH (2008). A new grading system evaluating bleeding scale in filament perforation subarachnoid hemorrhage rat model. J Neurosci Methods.

[33] Bouët V, Freret T, Toutain J, Divoux D, Boulouard M, Schumann-Bard P (2007). Sensorimotor and cognitive deficits after transient middle cerebral artery occlusion in the mouse. Exp Neurol.

[34] Zhang L, Schallert T, Zhang ZG, Jiang Q, Arniego P, Li Q, Lu M, Chopp M (2002). A test for detecting long-term sensorimotor dysfunction in the mouse after focal cerebral ischemia. J Neurosci Methods.

[35] Heindl S, Gesierich B, Benakis C, Llovera G, Duering M, Liesz A (2018). Automated morphological analysis of microglia after stroke. Front Cell Neurosci.

[36] Schneider UC, Davids AM, Brandenburg S, Müller A, Elke A, Magrini S, Atangana E, Turkowski K, Finger T, Gutenberg A, Gehlhaar C, Brück W, Heppner FL, Vajkoczy P (2015). Microglia inflict delayed brain injury after subarachnoid hemorrhage. Acta Neuropathol.

[37] Colonna M, Brioschi S (2020). Neuroinflammation and neurodegeneration in human brain at single-cell resolution. Nat Rev Immunol.

[38] Xiong XY, Liu L, Yang QW (2016). Functions and mechanisms of microglia/macrophages in neuroinflammation and neurogenesis after stroke. Prog Neurobiol.

[39] Zhang S (2019). Microglial activation after ischaemic stroke. Stroke Vasc Neurol.

[40] DI Vergouwen M, Vermeulen M, Coert BA, Stroes ES, Roos YB (2008). Microthrombosis after aneurysmal subarachnoid hemorrhage: an additional explanation for delayed cerebral ischemia. J Cereb Blood Flow Metab.

[41] Turan N, Heider RAJ, Zaharieva D, Ahmad FU, Barrow DL, Pradilla G (2016). Sex differences in the formation of intracranial aneurysms and incidence and outcome of subarachnoid hemorrhage: review of experimental and human studies. Transl Stroke Res.

[42] Luo Y, Reis C, Chen S (2018). NLRP3 inflammasome in the pathophysiology of hemorrhagic stroke: a review. Curr Neuropharmacol.

[43] Erdei J, Tóth A, Balogh E, Nyakundi BB, Bányai E, Ryffel B, Paragh G, Cordero MD, Jeney V (2018). Induction of NLRP3 inflammasome activation by heme in human endothelial cells. Oxidative Med Cell Longev.

[44] Wu X, Zhang H, Qi W, Zhang Y, Li J, Li Z, Lin Y, Bai X, Liu X, Chen X, Yang H, Xu C, Zhang Y, Yang B (2018). Nicotine promotes atherosclerosis via ROS-NLRP3-mediated endothelial cell pyroptosis. Cell Death Dis.

[45] Fann DYW, Lee SY, Manzanero S, Chunduri P, Sobey CG, Arumugam TV (2013). Pathogenesis of acute stroke and the role of inflammasomes. Ageing Res Rev.

[46] Yang F, Wang Z, Wei X, Han H, Meng X, Zhang Y, Shi W, Li F, Xin T, Pang Q, Yi F (2014). NLRP3 deficiency ameliorates neurovascular damage in experimental ischemic stroke. J Cereb Blood Flow Metab.

