# Supplementary figures and images for: NLRP3 inhibition attenuates early brain injury and delayed cerebral vasospasm after subarachnoid hemorrhage
Source: J Neuroinflammation. 2021 Jul 20;18:163. doi: 10.1186/s12974-021-02207-x (PMC8293512; doi:10.1186/s12974-021-02207-x)

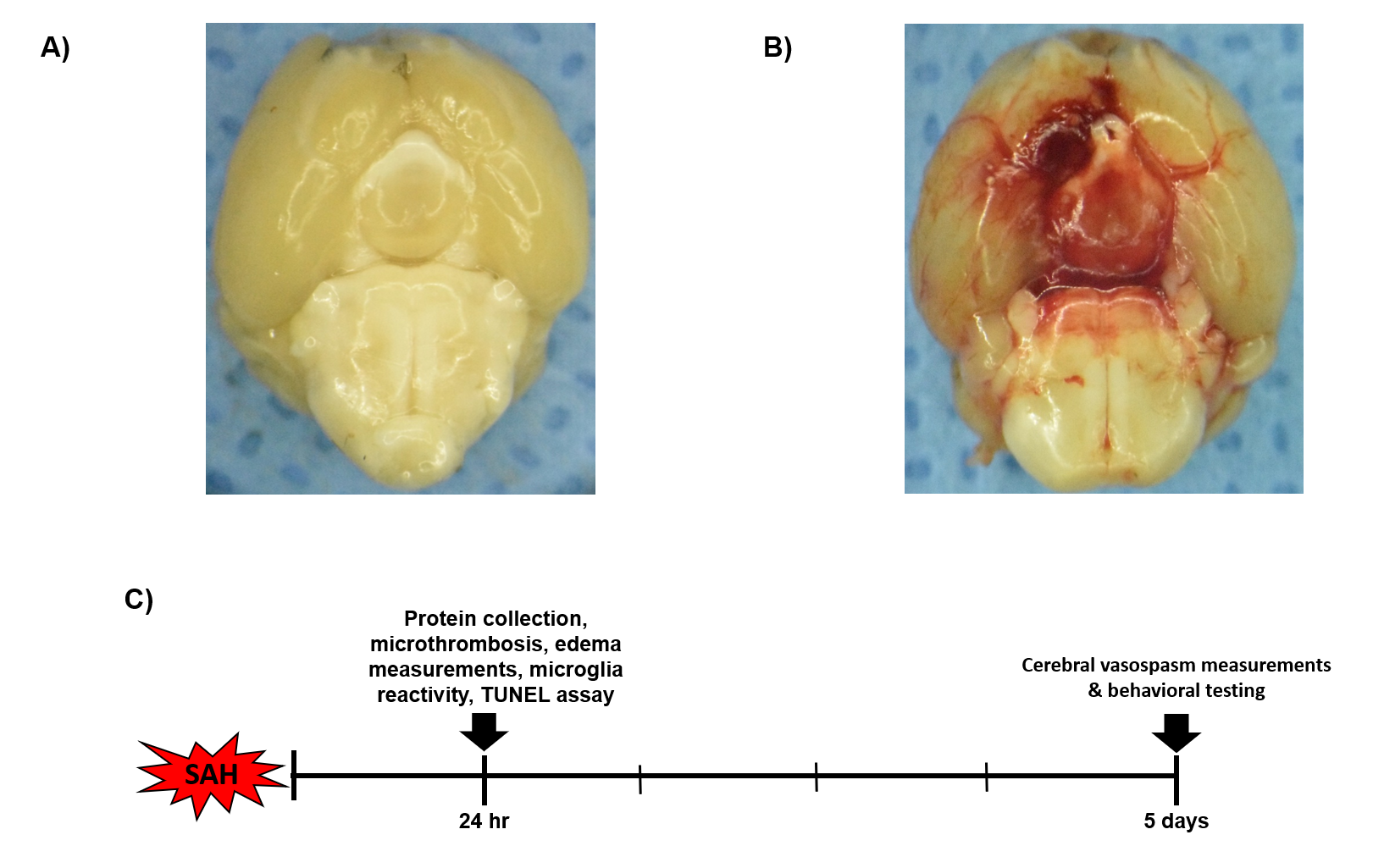

Supplement: Supplementary file 1 — Additional file 1: Supplemental Figure 1. A) Image of sham-operated brain immediately after surgery. B) Image of SAH-operated brain immediately after surgery. C) Experimental timeline. [file 12974_2021_2207_MOESM1_ESM.tif]

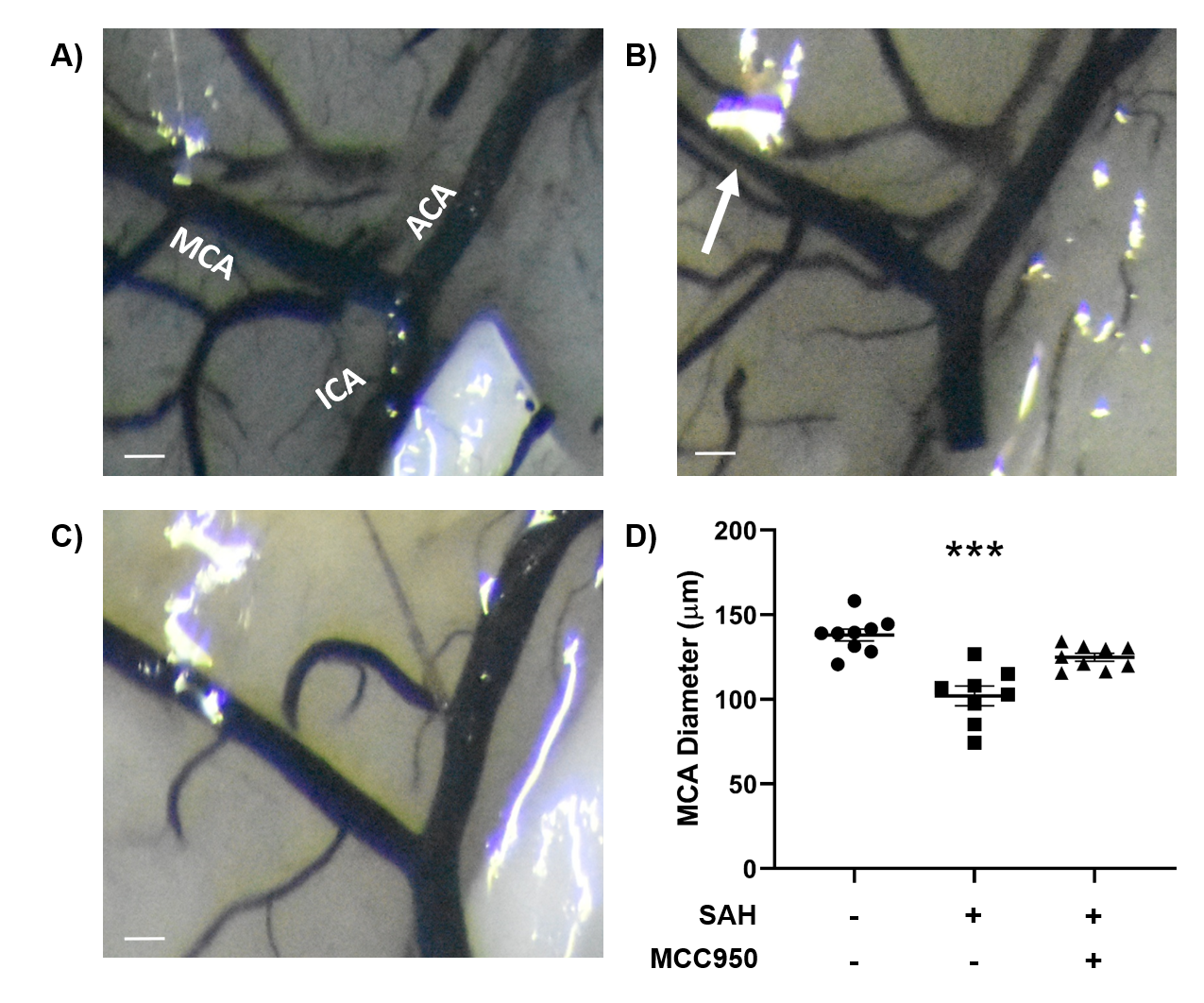

Supplement: Supplementary file 2 — Additional file 2: Supplemental Figure 2. Effect of MCC950 on cerebral vasospasm persists to seven days post-SAH. A-C) Representative images of vessel-casted brains of sham, SAH + vehicle, and SAH + MCC950 groups seven days after SAH. Internal carotid, middle cerebral, and anterior cerebral arteries are identified in sham image. Arrows indicate areas of significant vasospasm, scale bars = 100μm. D) Middle cerebral artery measurements normalized to sham group. Data presented as mean ± SEM, n = 8-9 per group, *** p < 0.001 compared to sham surgery group by Kruskal-Wallis test with Dunn’s multiple comparison. [file 12974_2021_2207_MOESM2_ESM.tif]
