# Supplementary material for: NLRP3 inhibition attenuates early brain injury and delayed cerebral vasospasm after subarachnoid hemorrhage
Source: J Neuroinflammation. 2021 Jul 20;18:163. doi: 10.1186/s12974-021-02207-x (PMC8293512; doi:10.1186/s12974-021-02207-x)
Supplement: Supplementary file 3 — Additional file 3. Raw blot files. [file 12974_2021_2207_MOESM3_ESM.pdf]

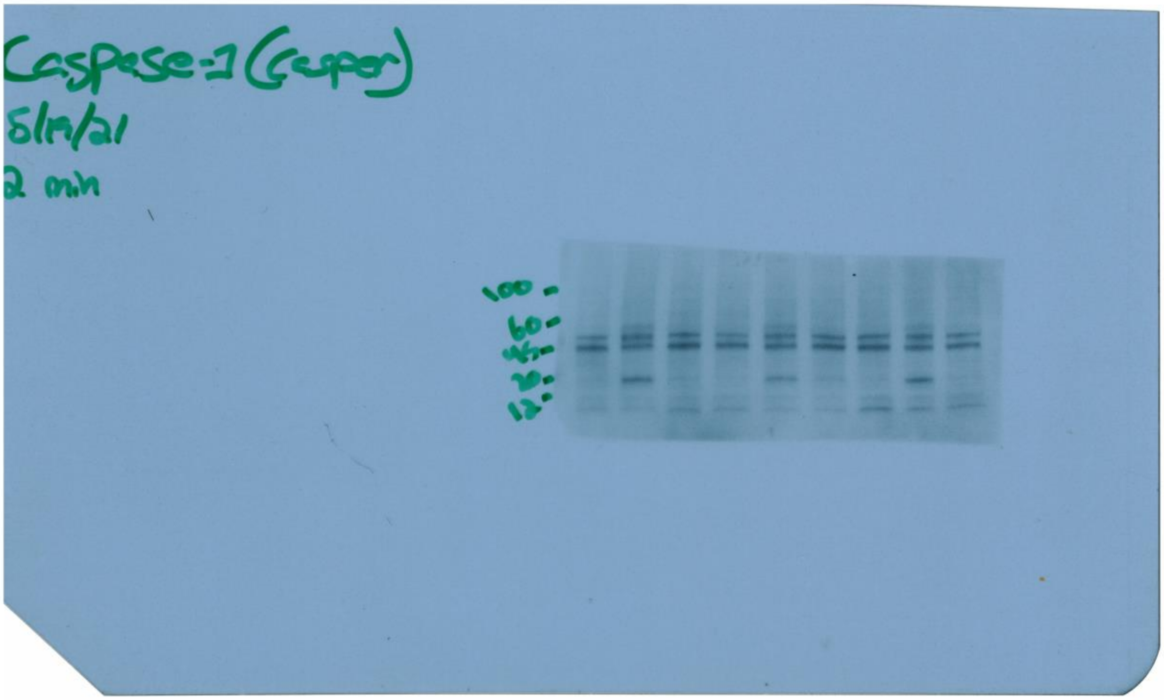
Caspase-1
2 min
100
60
45
12

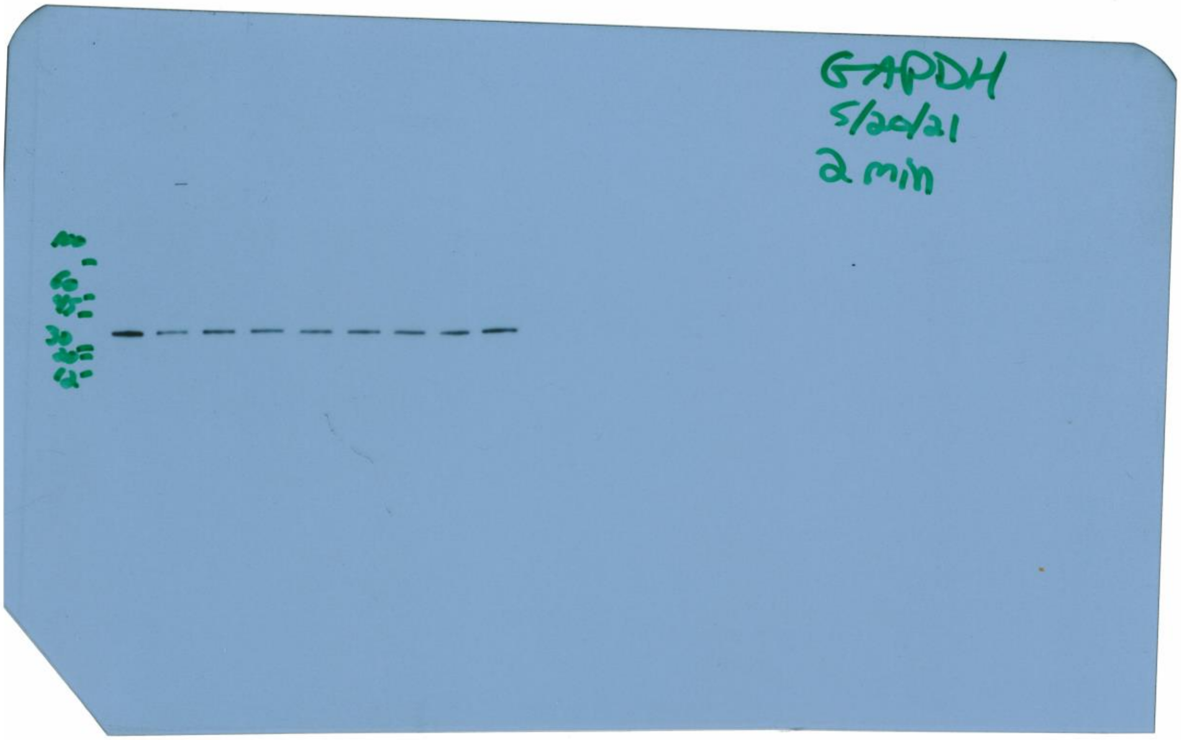
GAPDH
5/20/21
2 min

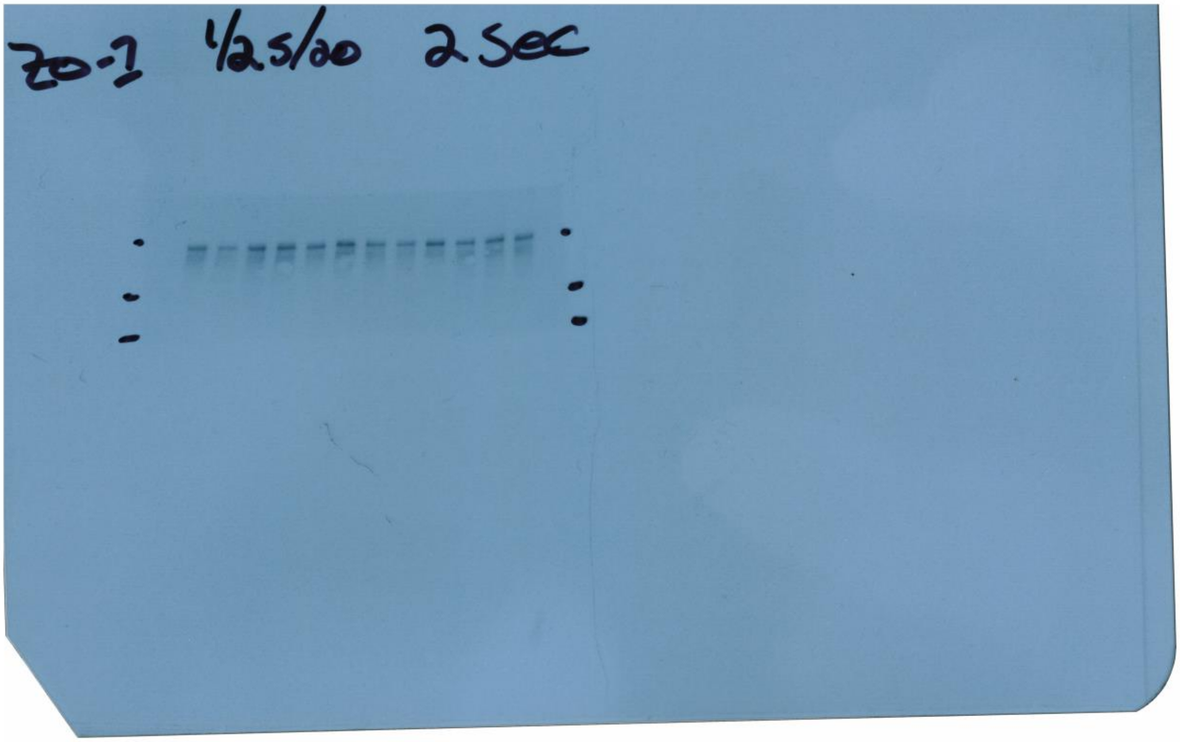
ZO-1 1/25/20 2 sec

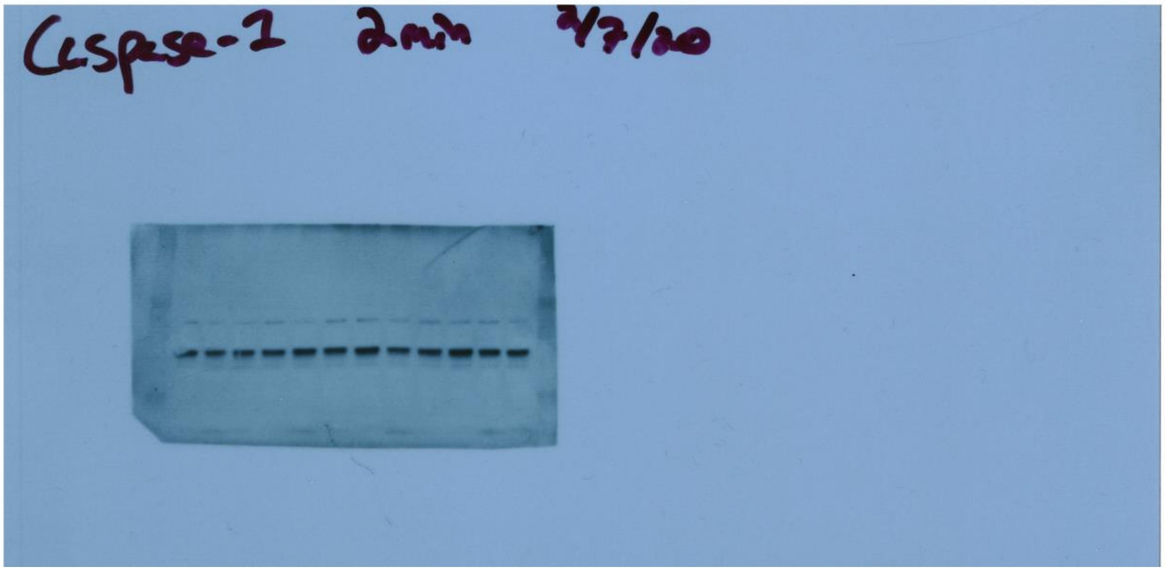
Caspase-1 2min 2/7/20

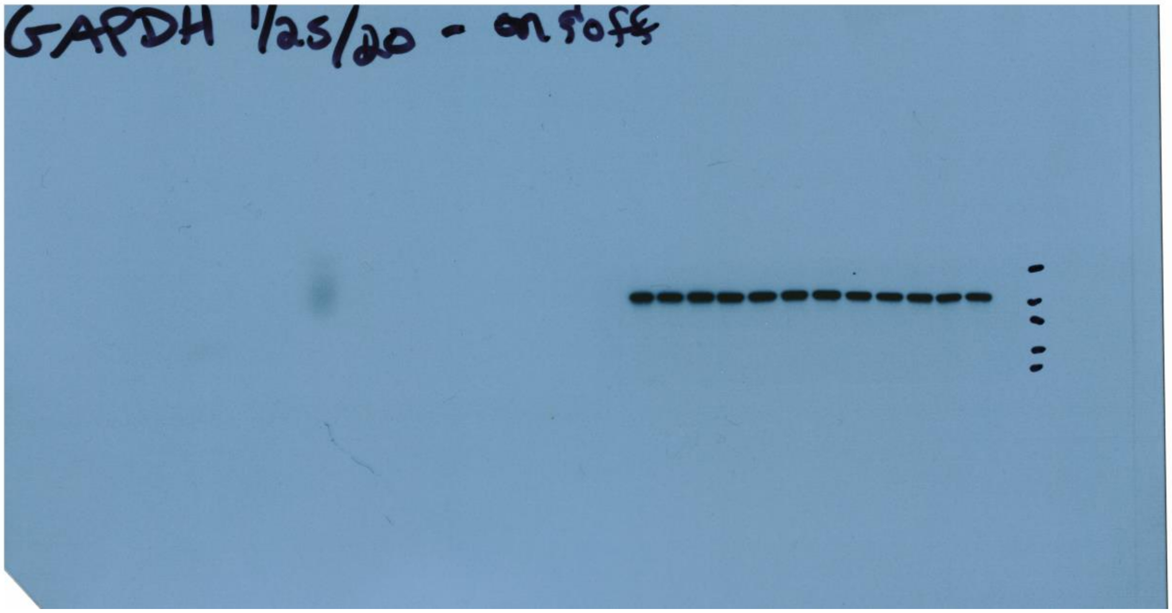
GAPDH 1/25/20 - on & off

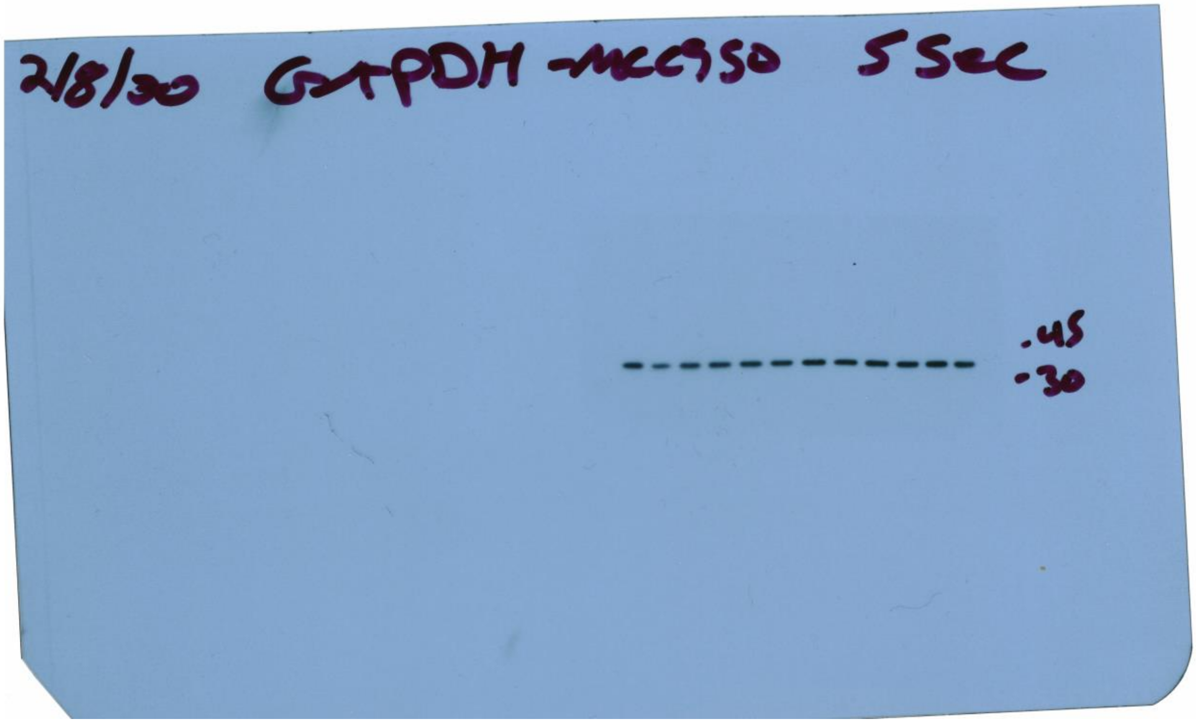
2/8/20 GAPDH -MCC950 5 Sec
.45
-30

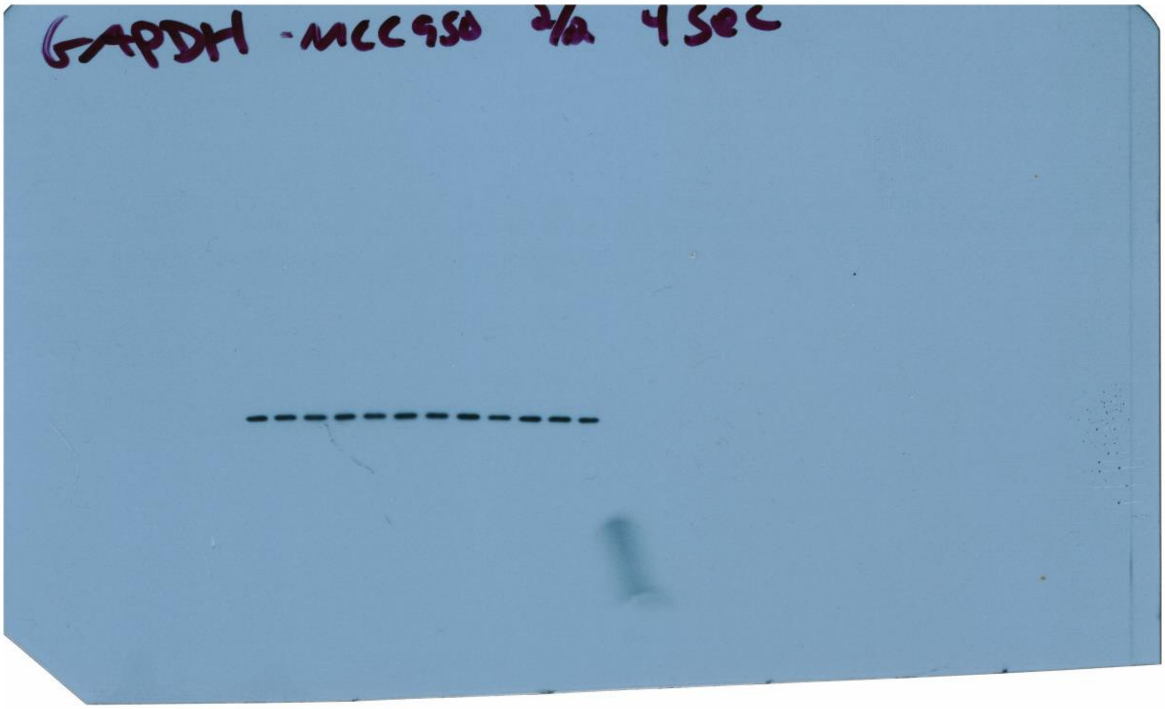
GAPDH -MCC950 2/12 4 sec

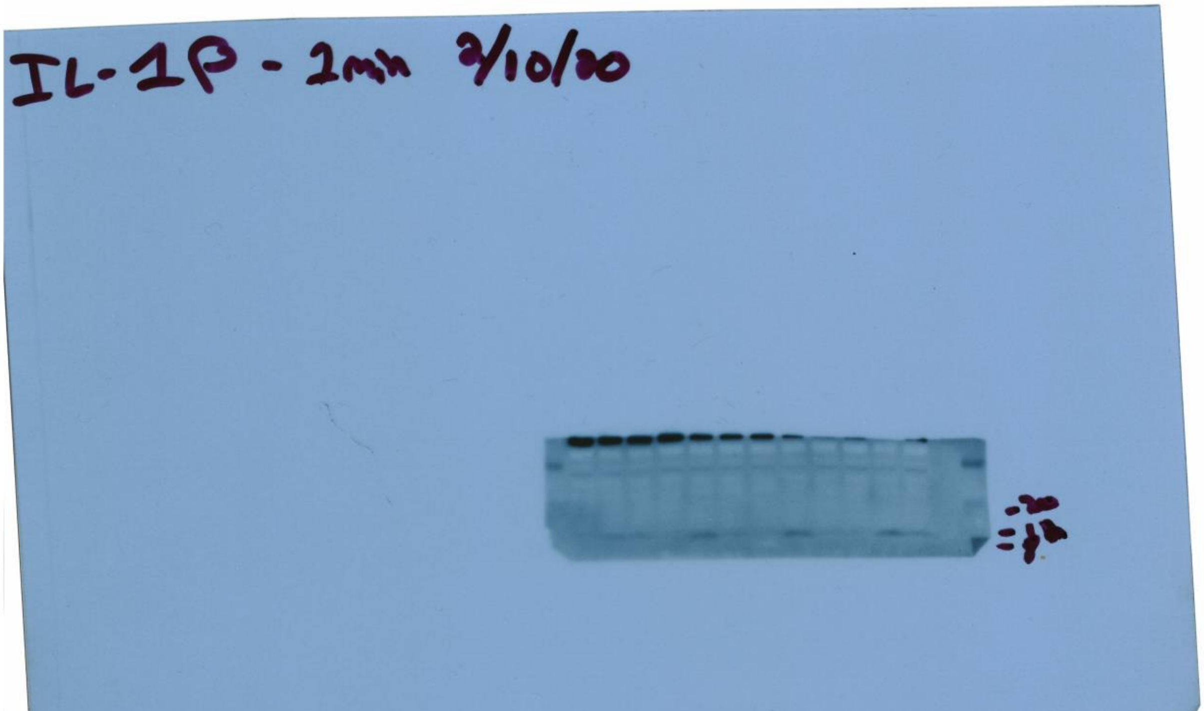
IL-1β - 2min 2/10/20
20
17

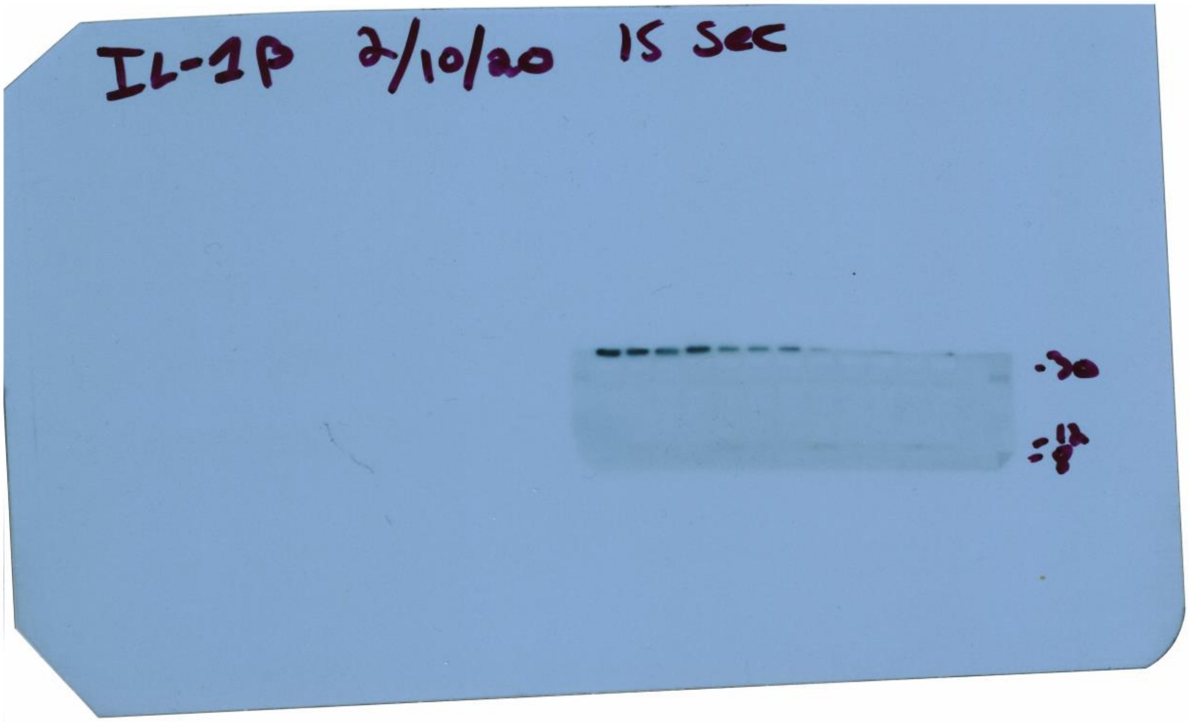
IL-1β 2/10/20 15 sec
30
12
8

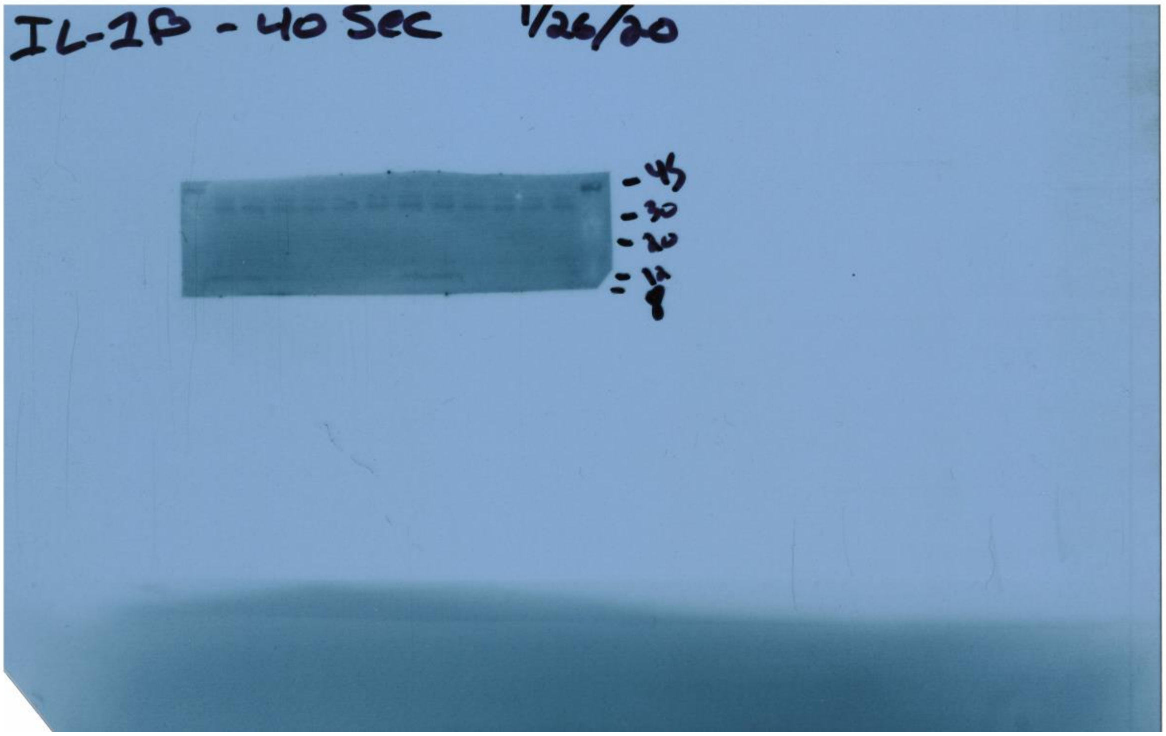
IL-1β - 40 sec 1/26/20
45
30
20
12
8

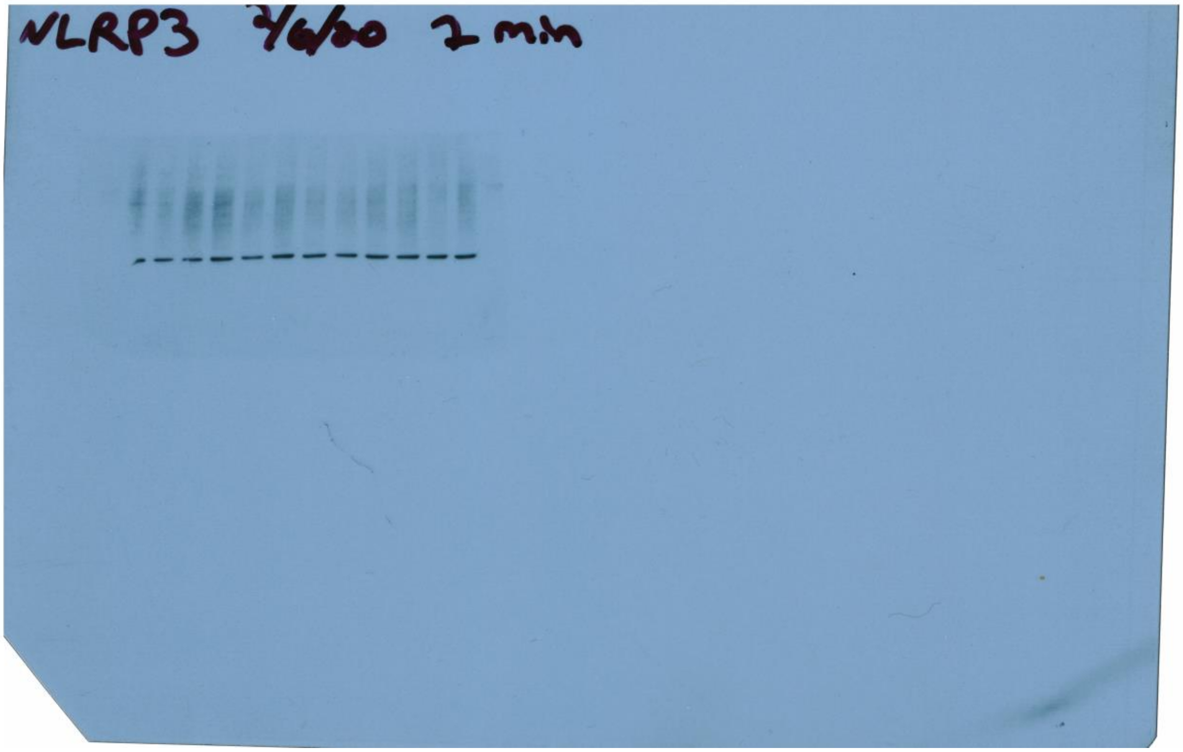
NLRP3 2/6/20 2 min

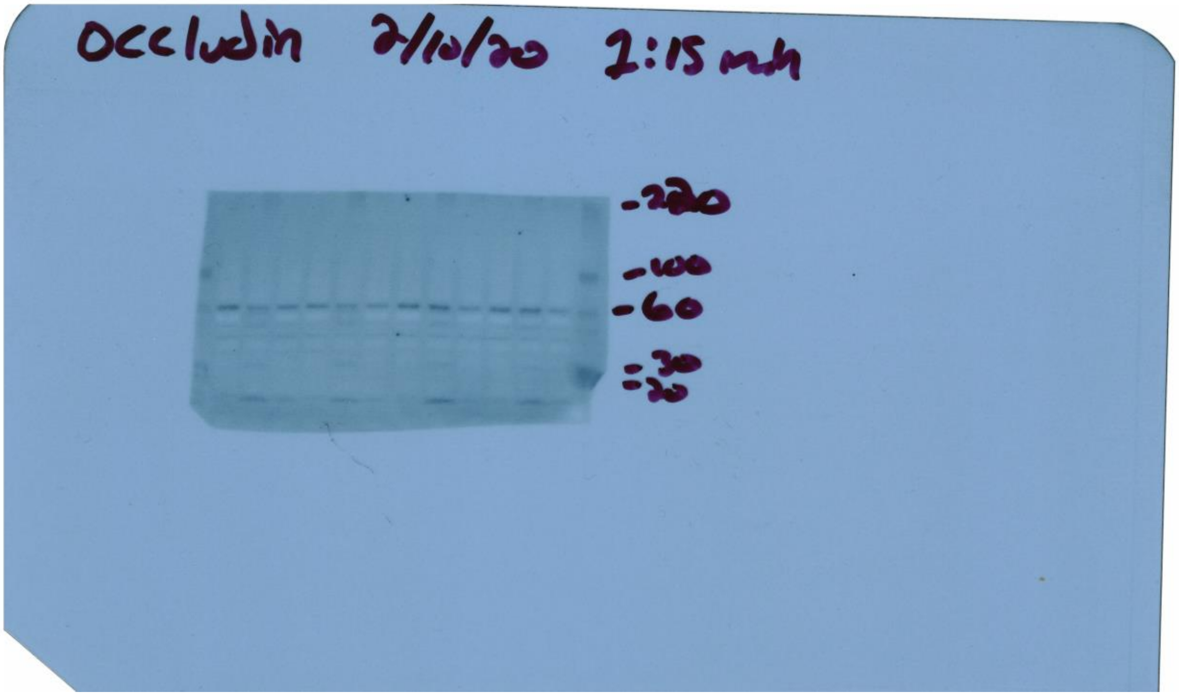
Occludin 2/10/20 2:15 min
-220
-100
-60
-30
-20
